# Estimation of glomerular filtration rate in conscious mice using a simplified equation

**DOI:** 10.14814/phy2.12135

**Published:** 2014-08-28

**Authors:** Yui Sasaki, Ryosuke Iwama, Tsubasa Sato, Kazuki Heishima, Shunsuke Shimamura, Tosihiro Ichijo, Hiroshi Satoh, Kazuhisa Furuhama

**Affiliations:** 1 Cooperative Department of Veterinary Medicine, Iwate University, Morioka, Iwate, Japan; 2 United Graduate School of Veterinary Science, Gifu University, Gifu, Japan

**Keywords:** Glomerular filtration rate, iodixanol, mice, plasma clearance, renal damage

## Abstract

To develop an expedient procedure for estimating glomerular filtration rate (GFR) in conscious mice, we first established a simple technique for repeated blood collections from the tail vein using a microcapillary tube attached to a 24‐gauge needle without the hub. Then, we devised a definition equation for estimation of the GFR using the contrast medium iodixanol as a test tracer. Iodixanol was administered as a bolus injection at 1500 mg I/kg to ddY mice, and the GFR was determined by the conventional multisample strategy. Based on cumulative data from the multisample method, an equation for the single‐blood‐sample method including the iodixanol dose, estimated distribution volume (Vd), and plasma iodixanol concentration at 60 min later was sought. The GFR values from the multisample method were in good agreement with those calculated using the equation. In clinically healthy mice, the GFR decreased gradually with ages from 11 weeks old in both sexes, suggesting the necessity of the corresponding control in each protocol. In nephropathy mice induced by cisplatin, the GFR values decreased with rises in serum BUN and creatinine concentrations, and serum creatinine became elevated when the GFR decreased to approximately 70% of the basal value. The results suggest that the simplified equation using iodixanol, allowing for the repeated use of the same mice, is a versatile procedure for research purposes.

## Abstract (precis)

To develop an expedient procedure for estimating glomerular filtration rate (GFR) in conscious mice, we first established a simple technique for repeated blood collections from the tail vein using a microcapillary tube attached to a 24‐gauge needle without the hub.

## Introduction

Measurement of glomerular filtration rate (GFR) is a gold standard for assessing kidney function and monitoring the progression of kidney disease in veterinary (Von Hendy‐Willson and Pressler 2011) and human (National Kidney Foundation 2002) medicine. However, classic urinary clearance method using the standard GFR tracer inulin is labor intensive because it requires constant infusion, and exactly repeated urine and blood collections in individuals. Moreover, inulin has an inherent disadvantage in nonlabeled formulas because of its extremely low solubility.

In mice, although isotopically or fluorescein isothiocyanate (FITC)‐labeled tracers have been most commonly utilized to date (Hackbarth and Hackbarth 1981; Qi and Breyer 2009; Schreiber et al. 2012), they need specific consideration and equipment. Thus, developing an easy, rapid, reliable, and practical procedure without surgical operation or anesthesia is essential for their laboratory investigations.

Recently, we reported the GFR determination in conscious rats using a single‐blood‐sample method with the isotonic, dimeric, nonionic iodine contrast medium iodixanol (Katayama et al. 2010) as a test tracer. Briefly, by substituting the GFR values and plasma iodixanol concentrations at a fixed time based on the data from the multisample method into Jacobsson's formula (Jacobsson 1983), the distribution of volume (Vd) was calculated. After confirming a relationship between the Vd values and plasma iodixanol concentrations, we obtained an equation for calculating the estimated Vd value. The GFR in the single‐blood‐sample method was obtained by substituting the iodixanol dose, estimated Vd value, sampling point, and plasma iodixanol concentration in each animal into Jacobsson's formula (Jacobsson 1983) once again. Because the estimated Vd value is dependent on elimination kinetics of each tracer and animal size, it is necessary to obtain it in the respective species (Katayama et al. 2010).

Jacobsson's formula (Jacobsson 1983) derived from the GFR calculation with one sample requires that the Vd value be known, and the accuracy in the Vd value determines the accuracy in the method. Similarly, if the Vd value of the tracer is known, the plasma disappearance curve can be closely approximated from a single, timed plasma measurement (Harvey et al. 1988).

Iodixanol used as the tracer is rapidly excreted into urine without degradation and no or very little protein binding in rats (Heglund et al. 1995; Jacobsen et al. 1995), monkeys (Heglund et al. 1995; Jacobsen et al. 1995), and humans (Svaland et al. 1992; Jacobsen et al. 1995), and can be easily and high sensitively detected by a high‐performance liquid chromatography (HPLC) with a small amount (10–20 *μ*L) of plasma sample. Additionally, iodixanol has been shown to be less nephrotoxic than other nonionic monomeric X‐ray contrast media in randomized, double‐blind, prospective, multicenter studies among patients with chronic renal diseases (Aspelin et al. 2003; McCullough et al. 2006; Karlsberg et al. 2010).

Here, we first established a simple technique for repeated blood collections from the tail vein of conscious mice, and then attempted to seek a definition equation based on Jacobsson's formula (Jacobsson 1983) with iodixanol for the GFR estimations. In this study, because the multisample strategy using iodixanol has been already established as a reliable one in rats (Katayama et al. 2010), we utilized the multisample method as the alternative standard procedure for estimating GFR. The highlight of this investigation is to be more efficient animal use.

## Methods

### Drugs

Iodixanol (Visipaque 320, 320 mg I/mL, 290 mOsm/kg H_2_O) was purchased from Daiichi‐Sankyo (Tokyo, Japan), and cisplatin (Cisplatin IV Infusion, 0.5 mg/mL) was provided from Yakult (Tokyo). The units for the dose level and plasma concentrations of iodixanol are milligram of iodine/kg body weight and microgram of iodine/mL, respectively. Cisplatin, a platinum chemotherapeutic agent, was used for preparing a severe kidney injury (Hanigan et al. 2001). All other chemicals and reagents were of the highest grade available from commercial sources, unless stated otherwise.

### Animals

All experimental procedures were performed in accordance with the Guidelines for Animal Experimentation issued by the Japanese Association for Laboratory Animal Science (Japanese Association for Laboratory Animal Science 1987) and also approved by the Animal Experimental Ethics Committee of Iwate University (No. A201139, Morioka, Japan).

Male and female Slc:ddY mice were obtained from Japan SLC (Shizuoka, Japan), and studies began after at least a 2‐day acclimation period. The ddY mice were chosen in this study because they were frequently used for toxicological screenings of chemical entities. The animals were housed in an air‐conditioned facility (temperature, 22 ± 3°C; relative humidity, 55 ± 25%; lighting, 8:00 am to 8:00 pm with a 12‐h light cycle) and fed commercial rodent chow (MEQ, Oriental Yeast, Tokyo, Japan) and tap water ad libitum.

### Blood collection

**Figure 1.**
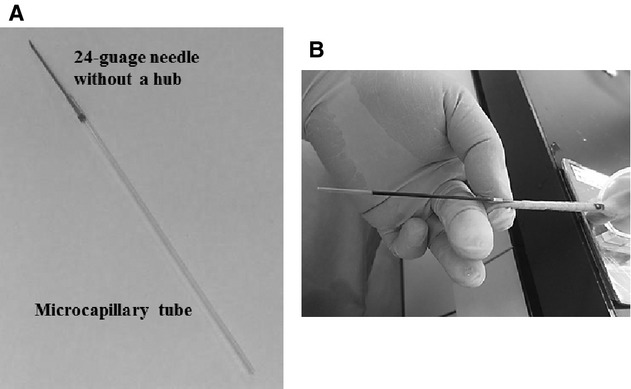
A microcapillary tube attached to a 24‐gauge needle without the hub (A), and blood collection technique from the tail vein of a conscious mouse (B).

The mouse was placed in a plastic restraining device (ICM‐3A, Ishizawa Corporation of Medical Implement, Tokyo, Japan). The tail was warmed to dilate the blood vessels with a light, and wiped with sterilizing cottons containing 70% ethanol. A 1‐inch long 24‐gauge needle, from which the hub was cut off by a Schwertfeile‐shaped metal file (Kougi, Niigata, Japan), was attached to a heparinized microcapillary tube (7.5 mm ×1.65 mm I.D., Terumo, Tokyo, Japan) mildly with an adhesive (Aron Alpha, Toagosei, Tokyo, Japan) (Fig. 1A). The needle was inserted into 1–2 cm of the distal part of the left tail vein at an angle of approximately 20° under the conscious conditions, and blood (approximately 40 *μ*L/time) was naturally withdrawn by capillary phenomenon (Fig. 1B). The bleeding was stopped by applying a small amount of the adhesive. After taking off the needle, the microcapillary tube containing blood was centrifuged at 15,000× g for 5 min with a centrifuge (MC‐201, Hitachi, Tokyo, Japan), and plasma was obtained by cutting a border between the supernatant and cell packages using the file.

### Appropriate iodixanol dose and blood sampling points

To determine the appropriate dose for the GFR measurement, iodixanol was administered intravenously at 750 or 1500 mg I/kg to the right tail vein of 12‐week‐old male mice (*n *=**4). Iodixanol was diluted with the 0.9% saline solution to be 150 or 300 mg I/mL, and administered at 5 mL/kg to each animal. Before the iodixanol injection, a constant volume (1 mL per mouse) of an isotonic mineral solution (Haruzen V injection, Nippon Zenyaku Kogyo, Koriyama, Japan) was orally given by gavage to all mice to avoid dehydration due to blood collection.

Blood was collected 30, 60, 90, 120, 150, and 180 min after the iodixanol injection under the conscious conditions by the aforementioned technique. To identify the proper blood sampling points, the GFR was calculated by various combinations of blood sampling points on the basis of the data from the above reference 1500 mg I/kg dose study. The combination of the sampling points for GFR estimations using a one‐compartment model was as follows: a) 60, 120, and 180 min later; b) 30, 60, 90, 120, 150, and 180 min later; c) 30, 60, 90, 120, and 150 min later; d) 30, 60, 90, 120, and 180 min later; e) 30, 60, 90, 150, and 180 min later; f) 30, 60, 120, 150, and 180 min later; g) 30, 90, 120, 150, and 180 min later; and h) 60, 90, 120, 150, and 180 min later. The sampling points of 60, 120, and 180 min later were selected based on the reported rat study (Katayama et al. 2010).

### Estimation of GFR in healthy and nephropathy mice

Iodixanol was administered intravenously at 1500 mg I/kg to healthy 8‐week‐old male mice (*n *=**10), and blood was collected 60, 120, and 180 min after the iodixanol injection. The GFR was estimated by the three‐blood‐sample method.

For the nephropathy model, cisplatin was administered intraperitoneally at a dose of 7.5 or 15 mg/kg to 8‐week‐old male mice. Control animals received 0.9% saline solution (saline, 30 mL/kg) in the same way. Four days later (day 5), the GFR was estimated by both the single‐ and three‐blood‐sample methods as mentioned below, and then all animals were euthanized by exsanguination under ether anesthesia. The first day of dosing was regarded as day 1 for this study. The cisplatin dose and observation periods used were selected from the results of the preliminary tests based on the previous report (Hanigan et al. 2001). These studies were performed thrice to confirm the reproducibility.

### Laboratory tests and renal pathology

Serum urea nitrogen (BUN: urease‐GLDH method) and creatinine (enzymatic method) concentrations were measured with an autoanalyzer (Accute TBA‐40FR, Toshiba Medical Systems, Tochigi, Japan). The right kidney from mice given cisplatin or saline was excised, fixed in 10% formalin, embedded in paraffin wax, cut at 3‐*μ*m thickness, stained with hematoxylin and eosin (H‐E), and histopathologically examined. The histological evaluation was conducted in a blinded manner.

### Analysis of plasma iodixanol concentrations

Plasma iodixanol concentration was measured with reversed‐phase HPLC according to a previously reported procedure (Jacobsen et al. 1995) with some modifications. Plasma specimen (10–20 *μ*L) was deproteinized by adding 20% trichloroacetic acid (Wako Chemicals, Tokyo, Japan) at a ratio of 1:1 and placed at 4°C for 30 min to complete precipitation before removal of the proteins by centrifugation (14,000 x g, 10 min, 4°C). The supernatant was centrifuged again under the same conditions. The HPLC system consisted of separation equipment (Alliance™ Waters 2690 Separations Module; Waters, Milford, MA), a UV detector (Waters 996 Photodiode Array Detector; Waters), and analytical software (Millennium^32^; Waters) equipped with a 250 × 4.6‐mm C‐18 reverse‐phase column (RP‐18 GP, 5 *μ*m; KANTO CHEMICAL, Tokyo, Japan). The stepwise mobile phase profile was composed of distilled water followed by 80% acetonitrile in distilled water, and the flow rate was maintained at 1 mL/min. The detection wavelength was 244 nm, which is the approximate absorbance maximum for iodixanol. The standard was prepared at known concentrations of iodixanol, and the results from the standard were used to calculate the concentration in each sample. The detection limit of plasma iodixanol concentration was 5 *μ*g I/mL. The intra‐ and interassay CVs in plasma iodixanol determination were 0.64 and 2.47%, respectively.

### Iodixanol clearance

Clearance calculation was based on the one‐compartment model. Briefly, the area under the plasma iodixanol concentration versus time curve (AUC) was calculated by the linear trapezoidal rule with extrapolation using three to six blood sampling points. A clearance value (*Cl*) was calculated from the following formula.



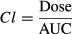



where dose is the dose level of iodixanol injected.

To estimate iodixanol clearance by the single‐blood‐sample method, the Vd value of iodixanol in each mouse was back‐calculated by substituting GFR values and plasma iodixanol concentrations (C_t_) at 60, 120, or 180 min (t) obtained from the multisample method using iodixanol into the following Jacobsson's formula (Jacobsson 1983).



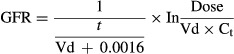



The above formula can be transformed into the following formula by the Newton method (Smale 1985; Varona 2002), and the “b” value which is a variable can be also solved.



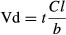



The Vd value was then reconfirmed by the “Goal‐Seek” function of Microsoft Office Excel 2007 (Microsoft Japan, Tokyo). To seek the estimated Vd in each mouse, after the correlation between the Vd value and C_t_ was assessed with an exponential regression, an equation for calculating Vd was determined. The Vd obtained from calculation was regarded as the estimated Vd in this study.

Finally, the GFR value by the single‐blood‐sample method using iodixanol was measured by substituting the estimated Vd value, the dosage level (dose, 1500 mg I/kg), and plasma iodixanol concentration (C_t_) at 60 min (t) from each animal into the above formula (eq. 2). The GFR is represented as *μ*L/min per g of body weight.

### Changes in GFR in mice with ages

To measure the GFR in mice with ages, iodixanol was administered intravenously at 1500 mg I/kg to clinically healthy male and female mice at 7 weeks of age (*n *=**10). Blood was collected at 60 min later, and the GFR was estimated by the single‐blood‐sample method. These estimations were repeatedly performed once weekly for eight consecutive weeks using identical mice (7–15 weeks old).

### Relationship between GFR values versus serum BUN or creatinine concentrations

The relationship between the GFR values from the single‐blood‐sample method versus serum BUN or creatinine concentrations was assessed using male and female mice (*n *=**75) at 7–15 weeks in preliminary and present studies.

### Statistical analysis

Quantitative data are expressed as the mean ± standard deviation (SD). Differences among more than three groups were compared using one‐way ANOVA and Dunnett's test. A *P* value of < 0.05 was considered statistically significant. Comparisons of the GFR values from the multisample method with those from the single‐blood‐sample method using iodixanol were performed according to a standard recommendation for comparing analytical techniques based on Deming's regression (Deming 1964) and Bland and Altman bias presentation (Bland and Altman 1986, 1999) using Prism 5 (GraphPad Software, San Diego, CA).

## Results

**Figure 2.**
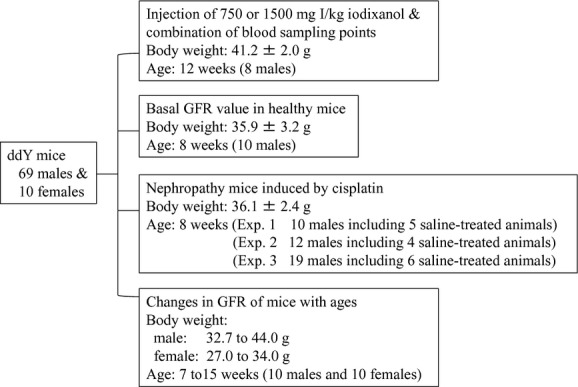
Experimental protocols used for this study.

The experimental protocol is summarized in Fig. 2 with body weight and age of mice used in the respective studies.

### Appropriate dose and blood sampling points of iodixanol for GFR estimations by the multisample method

**Figure 3.**
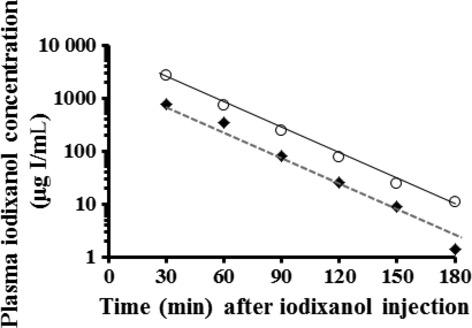
Representative disappearance of iodixanol from plasma after an intravenous administration of 750 (closed diamonds) or 1500 (open circles) mg I/kg iodixanol to 12‐week‐old male ddY mice. Blood was collected from the tail vein of identical mice under conscious conditions 30, 60, 90, 120, 150, and 180 min after iodixanol injection. Plasma iodixanol concentrations were measured by HPLC.

**Figure 4.**
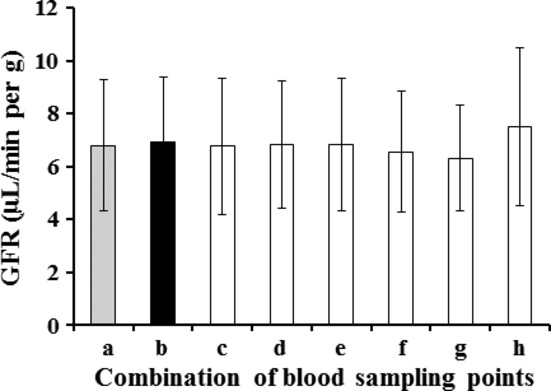
Glomerular filtration rate values estimated by combinations of various blood sampling points after an intravenous administration of 1500 mg I/kg iodixanol to 12‐week‐old male ddY mice; (a) 60, 120, and 180 min later (gray column); (b) 30, 60, 90, 120, 150, and 180 min later (black column); (c) 30, 60, 90, 120, and 150 min later; (d) 30, 60, 90, 120, and 180 min later; (e) 30, 60, 90, 150, and 180 min later; (f) 30, 60, 120, 150, and 180 min later; (g) 30, 90, 120, 150, and 180 min later; and (h) 60, 90, 120, 150, and 180 min later. Values represent the mean ± SD of four mice.

In healthy 12‐week‐old male mice given 750 or 1500 mg I/kg of iodixanol, mean concentrations of iodixanol in plasma had a linear disappearance until 180 min. At 750 mg I/kg, however, plasma concentrations decreased almost to the detection limit (5 *μ*g I/mL) 180 min after injection (Fig. 3). No difference was seen among the GFR values from three (6.81 ± 2.48 *μ*L/min per g), five (mean GFR ranges: 6.31–7.50 *μ*L/min per g), and six (6.91 ± 2.49 *μ*L/min per g) blood sampling points in the one‐compartment model with various combinations of sample points (Fig. 4).

### GFR estimated by the three‐blood‐sample method in healthy and cisplatin‐induced nephropathy mice

**Figure 5.**
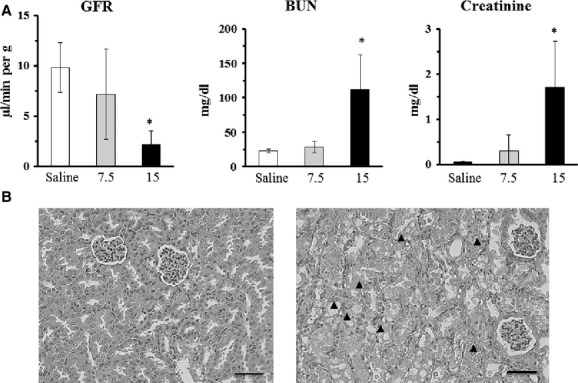
Glomerular filtration rate values, serum BUN and creatinine concentrations, and renal morphology on day 5 in 8‐week‐old male ddY mice given cisplatin. (A) Cisplatin was administered intraperitoneally at 7.5 (gray column) or 15 (black column) mg/kg. Mice given 0.9% saline solution (saline, 30 mL/kg) in the same way served as the control (open column). The GFR value was determined by the three‐blood‐sample method. Each column and vertical bar represents the mean ± SD of 6–7 animals. *P <**0.05 versus the saline group (Dunnett's test). (B) Necrosis (arrowheads) in the proximal epithelium and mitotic figures of epithelial cells in the proximal tubule were observed in mice receiving 15 mg/kg cisplatin (right), whereas no changes were noted in mice given saline (left). H–E staining, Bars = 100 *μ*m.

The background GFR values in healthy 8‐week‐old male ddY mice were 9.80 ± 1.01 *μ*L/min per g. In mice given intraperitoneally 15 mg/kg cisplatin, decreased GFR values (2.23 ± 1.30 *μ*L/min per g) were noted with significant increases in serum BUN (111.5 ± 51.1 mg/dL) and creatinine (1.80 ± 1.04 mg/dL) concentrations, although no changes were noted in mice given 7.5 mg/kg cisplatin (Fig. 5A). Renal histopathological examinations revealed that necrosis in the proximal epithelium and mitotic figures of epithelial cells in the proximal tubule were observed only in mice receiving 15 mg/kg cisplatin (Fig. 5B).

### GFR estimated by the single‐blood‐sample method

Using the cumulative data collected from the preliminary and present studies including healthy and nephropathy mice based on the multisample strategy, a formula for calculating the estimated Vd value was determined by a scatter diagram as follows;



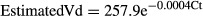



where Ct is the plasma iodixanol concentration at 60 min.

**Figure 6.**
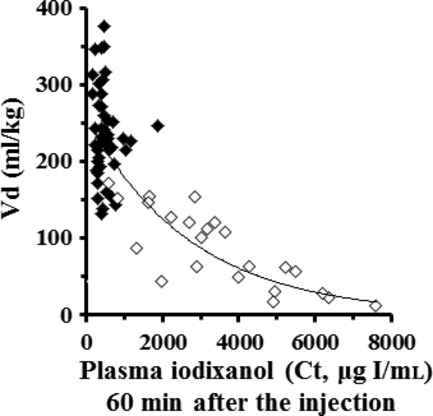
Scatter plots in volumes of distribution (Vd) and plasma iodixanol concentrations (Ct) 60 min after a bolus iodixanol administration to healthy (closed diamonds) and nephropathy (open diamonds) mice. Estimated Vd = 257.9e^−0.0004C^, *r *=**0.86 (*P *<**0.01). *n *=**65.

Plasma iodixanol concentration 60 min later (*r *=**0.86, *P *=**0.01, *n *=**65) was chosen as it showed an extremely high coefficient between Vd values and plasma iodixanol concentrations compared to that at 120 (*r *=**0.69) or 180 (*r *=**0.42) min later. The Vd values were 120–390 mL/kg and 10–170 mL/kg in healthy and nephropathy mice, respectively (Fig. 6).

**Figure 7.**
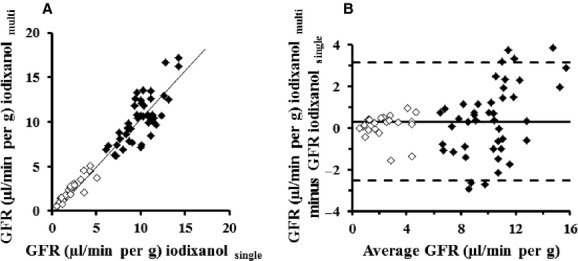
Plots of GFR estimation in male ddY mice with the single‐blood‐sample method (GFR _single_) compared with the three‐blood‐sample method (GFR _multi_) in healthy (closed diamonds) and nephropathy (open diamonds) mice. (A) Scatter plots between GFR _single_ and GFR _multi_. Deming regression was *y *=**1.08*x* − 0.47 (*r *=**0.90). (B) Bland–Altman representation of the differences between the two methods according to the GFR measured. Mean bias (solid line): 0.30. Upper and lower values represent 95% limit of agreement plots: mean bias ± 2.83 (dotted lines). *n *=**65.

A correlation was noted between the GFR values estimated by the multisample and single‐blood‐sample methods using iodixanol (*r *=**0.90, *P *<**0.001, *n *=**65, Fig. 7A). On the basis of Bland and Altman bias presentation, approximately 91% (59/65 samples) was within the agreement plots, although six points were outliers (Fig. 7B).

### Changes in GFR in mice with ages

**Figure 8.**
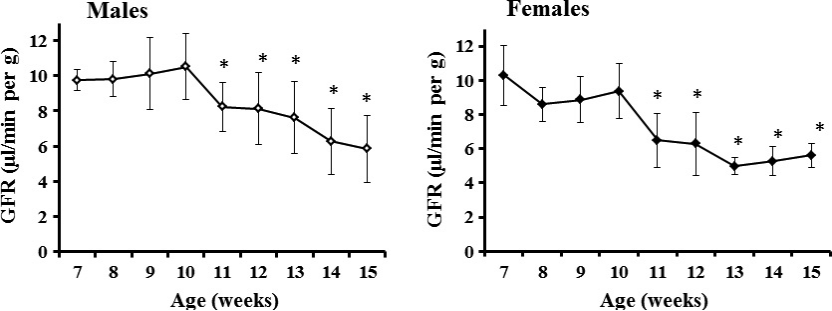
Age‐related profiles of glomerular filtration rate (GFR) in clinically healthy male and female ddY mice under the conscious conditions. The GFR was estimated by the single‐blood‐sample method. Values represent the mean ± SD of 10 animals. *P <**0.05 versus mice at 8 weeks (Dunnett's test).

The GFR in clinically healthy male and female mice remained stable by 10 weeks, and then decreased gradually from 11 weeks onward (Fig. 8). No sex difference was seen under the present experimental conditions.

### Relationship between GFR values versus serum BUN or creatinine concentrations

**Figure 9.**
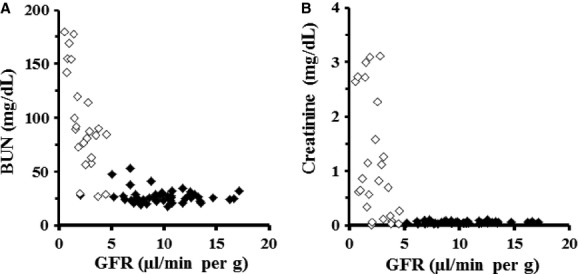
Relationship between the GFR values from the single‐blood‐sample method versus serum BUN (A) or creatinine (B) concentrations using the data collected from healthy (closed diamonds) and nephropathy (open diamonds) mice. *n *=**75 including both sexes.

When the GFR value decreased to more than 70% of the mean basal level (11.5 *μ*L/min per g) in mice at 7–10 weeks, serum creatinine concentrations likely began to increase (Fig. 9B), whereas serum BUN concentration exhibited fluctuations to some extent (Fig. 9A).

## Discussion

In conscious mice, although there are a number of efficient methods available on collecting blood (Hoff 2000), the tail vein is preferably used for serial blood collection as often as need. In this work, we first established a simple technique for repeated blood collections from the tail vein using the microcapillary tube attached to a 24‐gauge needle to minimize pain and stress. Using this technique, we were able to collect the blood sample of approximately 40 *μ*L within 30 s, even in repeated blood withdrawals.

In multisampling strategies for mouse GFR measurement, the appropriate iodixanol dose and blood sampling points were first determined. Intravenous treatment of mice with 1500 mg I/kg iodixanol resulted in a linear semilogarithmic plot of plasma iodixanol concentrations versus time, demonstrating the suitability of using the one‐compartment model. Moreover, no significant difference was found among the GFR values estimated from three, five, and six blood sampling points (Fig. 4). This kinetics was a simplification and applied only after an equilibration period (elimination phase) compared to the two‐compartment model. Although the AUC calculated by the two‐compartment model was considered to be more exact (and somewhat higher) values than that by the one‐compartment model, it is extremely hard to perform repeated blood collections from an identical mouse technically at the distribution phase shortly after iodixanol injection. Moreover, Jacobsson's equation was developed originally based on the one‐compartment model. Therefore, the one‐compartment model with a combination of 1500 mg I/kg iodixanol and blood sampling points of 60, 120, and 180 min later was selected for subsequent multisample studies. The advantage of the condition with three sampling points was that a laboratory experiment using 2–4 groups of 5–10 mice each could be easily performed at a time interval difference mode.

The mean basal GFR values (9.80 *μ*L/min per g) in 8‐week‐old male ddY mice determined by the three‐blood‐sample method nearly resembled the reference data reported previously (Hackbarth and Hackbarth 1981; Qi and Breyer 2009; Schreiber et al. 2012), although the experimental conditions were very different.

In mice treated with 15 mg/kg cisplatin, severely decreased GFR values along with marked increases in serum BUN and creatinine concentrations were linked to devastating changes in the renal morphology. These results were well consistent with the reported results (Hanigan et al. 2001).

The Vd value in an individual mouse was determined by substituting the GFR value and plasma iodixanol concentration at 60 min obtained from the three‐blood‐sample method into Jacobsson's formula (Jacobsson 1983). The GFR values calculated at 60 min were apparently stable in mice compared to those at 120 or 180 min, because a relatively high concentration at 60 min or an extremely low concentration at 180 min was included. It is crucial to have a close relationship between the Vd value and plasma iodixanol concentration as a prerequisite for a definition equation to calculate the estimated Vd value. Generally, an agent possessing the Vd value with less than 600 mL/kg is considered to be in the extracellular fluid (interstitial volume outside the vessels), if it distributes to plasma and organs within a split second after injection (Ritschel 1986). As the estimated Vd of iodixanol was less than 400 mL/kg in both healthy and nephropathy mice, it was considered to exist in the blood stream or the extracellular fluid, but not in the intracellular fluid (Ritschel 1986). The estimated Vd values obtained closely resembled the apparent Vd value (280 mL/kg) reported in healthy volunteers (Svaland et al. 1992). Thus, the concept (Svaland et al. 1992; Heglund et al. 1995; Jacobsen et al. 1995) that nonionic iodine was not transferred to the tissues may be partially proven by these Vd values.

In Bland and Altman bias presentation, the GFR values were frequently within the agreement plots between the two methods, although the causes of six outliers remained unclear (Fig. 7b). This suggests that the single‐sample method can be used to estimate the GFR in mice as an alternative to the three‐blood‐sample method.

The GFR values in healthy mice remained stable by 10 weeks, and then decreased gradually from 11 weeks onward (Fig. 8), although there was no sex difference with the protocol used. If this procedure is applied to laboratory studies with mice, a corresponding control group would be essential for each protocol.

Based on the cumulative data obtained by the equation in healthy and nephropathy mice, serum BUN concentration exhibited large fluctuations (Fig. 9A), because BUN was affected by extrarenal factors including food intake and reabsorbed in the proximal tubules. Meanwhile, serum creatinine concentrations likely began to increase from the point at which the GFR values decreased to more than 70% of the mean basal level (Fig. 9B). Recently, 35–50% of the excreted creatinine in mice were reported to be secreted by the tubules rather than filtered by the glomeruli (Eisner et al. 2010). In fact, the basal serum creatinine levels (0.10 mg/dL or so) of healthy ddY mice were extremely lower than those (0.5–1.0 mg/dL) of rats (Katayama et al. 2010), cats (Polzin et al. 2005), dogs (Polzin et al. 2005), and humans (National Kidney Foundation 2002). The findings implied that serum BUN and creatinine concentrations may be a blunt marker of kidney function in mice.

Further investigations are required to collect cumulative background data including strain differences and various diseased models. Moreover, it remains to be established whether the equation is practically advantageous.

In conclusion, the single‐blood‐sample method with a bolus injection of iodixanol, allowing for the repeated use in the identical mice, is a versatile procedure without using surgical procedure, specific equipment, or anesthesia.

## Acknowledgments

We would like to thank Dr. Teturo Yamashita (Iwate University, Morioka, Iwate, Japan) for his advice and suggestions on measuring plasma iodixanol concentration with HPLC. We also acknowledge Dr. Yoji Furuhama (former NASDA, Tokyo, Japan) for providing information on the Newton method.

## Conflict of Interest

None of the authors of this article has a financial or personal relationship with other people or organizations that could inappropriately influence or bias the content of this study. 
